# From Awareness to Action: Nurses’ Experiences of Infection Control Measures in Clinical Practice

**DOI:** 10.1155/jonm/2038990

**Published:** 2026-06-25

**Authors:** İlknur Özkan, Cansu Polat Dunya, Nurdane Guder, Meryem Salhaoglu, Fatma Karataş Erbay

**Affiliations:** ^1^ Kumluca Faculty of Health Sciences, Internal Medicine Nursing Department, Akdeniz University, Kumluca, Antalya, Türkiye, akdeniz.edu.tr; ^2^ School of Nursing, Internal Medicine Nursing Department, Istanbul University, Istanbul, Türkiye, istanbul.edu.tr; ^3^ Faculty of Medicine, Internal Medicine Department, Istanbul Unıversity, Istanbul, Türkiye

**Keywords:** healthcare-associated infections, infection control, nurse, qualitative

## Abstract

**Aim:**

This study aimed to explore nurses’ experiences with the implementation of infection control measures, with a particular focus on perceived barriers, compliance practices, and organizational support mechanisms.

**Background:**

While infection control is a critical component of patient safety, the practical implementation of infection control protocols often encounters systemic and environmental barriers, particularly in inpatient care settings.

**Methods:**

A qualitative study was conducted using the interpretative phenomenological analysis (IPA) approach. Semistructured interviews were conducted online via Zoom with 15 nurses working in internal medicine units at a university hospital in Türkiye between September and December 2024. Each participant had at least 2 years of professional experience. Data were analyzed inductively in line with IPA principles to identify recurring themes and meaning structures.

**Results:**

Four main themes emerged: (1) Awareness and Compliance, reflecting nurses’ knowledge and routine practices related to infection control; (2) Challenges in Implementation, including workload pressures, staffing shortages, and physical environment limitations; (3) The Role of Training, emphasizing the perceived value of interactive and practice‐based education; and (4) Monitoring and Continuous Improvement, highlighting the need for regular audits and constructive feedback mechanisms. Notably, the barriers identified were predominantly organizational and systemic rather than individual, underscoring the importance of institutional support.

**Conclusion:**

Although nurses demonstrated strong awareness and commitment to infection prevention, structural and managerial challenges appeared to limit the consistent implementation of infection control measures. Strengthening institutional support, revising audit mechanisms, and enhancing staff engagement may help support sustainable infection control practices.

**Implications for Nursing Management:**

Nurse leaders and healthcare administrators may consider addressing workforce capacity, supporting practice‐oriented training approaches, and implementing constructive supervision and feedback systems to enhance adherence to infection control standards and promote a culture of safety in clinical practice.

## 1. Introduction

Hospital‐acquired infections (HAIs) remain a major challenge in contemporary healthcare and continue to threaten patient safety and public health worldwide [1, 2]. They are especially prevalent in high‐risk settings, including intensive care and internal medicine units, where patients are often immunocompromised and exposed to invasive procedures. In addition to increasing morbidity and mortality, HAIs prolong hospital stays and create a substantial economic burden for healthcare systems [3–5].

The consistent implementation of infection control measures is one of the most effective strategies for preventing HAIs and maintaining care quality [6]. Institutional policies and clinical guidelines provide the necessary framework; however, their effectiveness depends largely on how well frontline healthcare professionals integrate these measures into daily practice [7, 8]. In the context of this study, infection control measures refer to routine clinical practices such as hand hygiene, the use of personal protective equipment (PPE), isolation precautions, aseptic techniques during invasive procedures, and environmental hygiene measures commonly applied in internal medicine settings.

Nurses occupy a central position in infection prevention because of their continuous and direct involvement in patient care. As the healthcare professionals most consistently present at the bedside, they are the key to implementing infection control measures and sustaining patient safety [9–11]. Infection control practice, however, cannot be understood solely as a matter of knowledge or individual compliance. Current evidence indicates that adherence is shaped by behavioral, organizational, and contextual influences. Recent studies have shown that nurses’ knowledge, attitudes, practices, and perceived barriers related to infection control vary across clinical contexts, particularly in high‐acuity areas such as intensive care and care of patients receiving mechanical ventilation [12–15]. Related findings also suggest that clinical decision‐making in time‐critical conditions such as sepsis is influenced by system pressures and training gaps, indicating that knowing what to do does not always translate into consistent bedside implementation [16].

Despite these insights, the day‐to‐day experience of implementing infection control measures in routine clinical care remains insufficiently understood. Existing studies have focused mainly on knowledge levels, compliance rates, or institutional strategies, while fewer have examined how nurses interpret and negotiate infection control demands under conditions of workload, staffing shortages, material constraints, and team dynamics [17–19]. This gap is particularly critical in internal medicine clinics, which are characterized by high patient turnover, complex and multimorbid patient profiles, and prolonged hospital stays that increase exposure to infection risk. Evidence indicates that delayed discharge and high workload in internal medicine settings contribute to increased care complexity and pressure on healthcare professionals [20]. In addition, infection prevention practices are shaped not only by individual knowledge but also by system‐level and human factors, including workflow intensity, interprofessional dynamics, and environmental conditions [8, 21, 22]. These interacting factors may influence adherence in ways that are not fully captured by quantitative or purely descriptive approaches.

An idiographic exploration of nurses’ lived experiences is therefore valuable for understanding infection control practices in clinical settings. An interpretative phenomenological perspective allows exploration beyond whether nurses comply with protocols, making it possible to examine how they make sense of these practices, the tensions they experience in sustaining them, and the meanings they attribute to organizational barriers and support mechanisms. Such an approach can help explain the gap between institutional expectations and clinical realities while generating context‐sensitive knowledge to inform feasible, setting‐specific interventions.

This study explores how nurses working in internal medicine clinics experience the implementation of infection control measures in daily practice. It aims to explore and interpret how nurses make sense of the barriers they encounter, the strategies they employ, and the institutional conditions that shape their practices. By examining infection control through the lens of lived experience, the study seeks to contribute practical and contextually grounded insights for strengthening infection prevention practices and informing nursing management.

## 2. Materials and Methods

### 2.1. Study Design

This study employed an interpretative phenomenological analysis (IPA) approach to understand the experiences of nurses working in internal medicine clinics regarding the implementation of infection control measures. The IPA methodology, rooted in phenomenology, was selected to explore how nurses make sense of their lived experiences with infection control in complex clinical environments, consistent with established IPA methodological principles [23]. This approach is particularly appropriate when the goal is to understand meaning‐making and subjective interpretation within a real‐world healthcare context. In line with IPA’s double hermeneutic nature, as described by Smith [24], the researcher plays an active interpretive role, attempting to make sense of how participants themselves make sense of their experiences. To ensure reflexivity within this interpretive process, reflexive journaling was maintained throughout data collection and analysis, allowing the researcher to critically reflect on potential assumptions, preconceptions, and their potential influence on data interpretation.

### 2.2. Theoretical Framework

Interpretative phenomenology is a qualitative research approach that seeks to understand individuals’ lived experiences of a particular phenomenon and to uncover the meaning attributed to these experiences [23]. This study focused on how nurses perceive infection control measures and the difficulties they encounter in applying them. The IPA method facilitates the exploration of subjective experiences and yields insights into the meanings participants attach to everyday clinical challenges and strategies. This method is particularly suitable for capturing nuanced perspectives within clinical practice.

### 2.3. Study Setting and Recruitment

The study was conducted in the internal medicine clinics of a large tertiary‐level university hospital in Türkiye. The hospital is a high‐capacity teaching and research institution with approximately 150 inpatient beds in the internal medicine department, providing advanced healthcare services across multiple specialized units with high patient turnover. These clinics are characterized by high patient traffic and complex patient profiles, which combine to create an environment where the risk of infection is particularly high. The research setting offers a real‐world context where the implementation of infection control measures is of critical importance. This study was carried out in a natural setting, enabling the examination of nurses’ daily practices and the challenges they encounter related to infection control measures. The environment offers valuable insight into the daily barriers and practices of nurses, emphasizing the importance of infection prevention in high‐risk clinical areas.

Participants were initially approached via institutional email and later contacted individually by phone to confirm their interest and schedule interview appointments. All interviews were conducted online using Zoom. No one else was present during the interviews aside from the participant and the researcher. To ensure confidentiality in the online setting, participants were asked to join the interviews from a private and quiet location and to use personal devices. Headphones were recommended to prevent unintended disclosure of audio content. All Zoom sessions were conducted using password‐protected meetings and were not cloud‐recorded; instead, recordings were stored securely on a password‐protected institutional computer accessible only to the research team.

### 2.4. Inclusion and Exclusion Criteria

Participants were eligible if they were registered nurses working in internal medicine clinics, had at least 2 years of clinical experience, were directly involved in patient care, and had experience with routine infection control practices in their daily work. Nurses who were on leave during the data collection period, worked only in administrative roles, or had no direct clinical responsibilities were excluded.

Purposive sampling was employed to recruit participants who were considered information‐rich cases and able to provide detailed accounts of infection control implementation in internal medicine settings [25]. Variation in age, gender, and years of professional experience was considered to capture diverse perspectives within the study context.

The sample consisted of 15 nurses (11 women and 4 men), aged between 27 and 48 years, with clinical experience ranging from 2 to 24 years (Table 1). The sample size was deemed sufficient as data saturation was achieved—no new themes emerged during the later stages of data collection. No participants refused to participate or dropped out of the study. All nurses who were invited and met the inclusion criteria agreed to be interviewed.

**TABLE 1:** Demographic characteristics of participants.

Participant ID	Age (years)	Gender	Years of experience	Educational level
1	29	Female	5	Bachelor’s
2	34	Male	8	Bachelor’s
3	41	Female	12	Master’s
4	28	Female	4	Bachelor’s
5	36	Female	9	Bachelor’s
6	45	Female	15	Master’s
7	30	Male	6	Bachelor’s
8	39	Female	11	Master’s
9	33	Female	7	Bachelor’s
10	42	Male	13	Master’s
11	27	Female	3	Bachelor’s
12	38	Male	10	Bachelor’s
13	32	Female	6	Bachelor’s
14	44	Female	16	Master’s
15	40	Female	14	Master’s

### 2.5. Data Collection

Data were collected through semistructured, in‐depth interviews conducted online via Zoom. Each interview lasted approximately 45–60 min. The interview guide included themes such as general experiences with infection control, perceived barriers, coping strategies, and the impact of training. Example questions included “Can you describe your experiences with implementing infection control measures in your daily clinical practice?,” “Can you tell me about a situation in which you found it difficult to apply infection control measures?,” “How do you respond to or manage these challenges?,” and “In what ways do training and institutional factors influence your infection control practices?.” The guide was informed by the literature and expert opinion and was pilot tested for clarity and relevance. No repeat interviews were conducted. All interviews were audio‐recorded with participants’ consent, and field notes were taken immediately after each session to capture contextual observations and preliminary reflections. To ensure open dialog, researcher interaction during interviews was kept to a minimum. This approach encouraged participants to share their experiences freely and honestly. Data saturation was reached by the 13th interview, and two additional interviews were conducted to confirm that no new themes emerged. Transcripts were not returned to participants for additional comment; however, member checking was performed during the data analysis phase by sharing the identified themes with participants for validation. All interviews were conducted by the first author, who is a PhD‐level researcher and a registered nurse with professional experience in infection control and qualitative research. At the time of the study, she was working as an academic in internal medicine nursing at a university faculty. Her background includes formal training in qualitative methods and practical involvement in infection prevention initiatives. The researcher is a female and had no prior relationship with the participants.

### 2.6. Data Analysis

Data were analyzed using the IPA approach, as outlined by Smith et al. [23]. This method facilitates an exploration of participants’ lived experiences and seeks to uncover the underlying meanings they attribute to those experiences. The analytical process followed several systematic stages, in line with established IPA analytical procedures [23, 24]. First, interview transcripts were subjected to detailed, line‐by‐line coding, as recommended in IPA analytical practice [24]. Key statements were identified, highlighted, and coded independently to enhance analytical rigor and credibility.

Discrepancies between coders were discussed in regular consensus meetings, and where agreement could not be reached, a third researcher was consulted to resolve differences.

Subsequently, a coding tree was constructed by clustering initial codes into broader categories, which were then further abstracted into overarching themes. In total, 128 initial codes were generated from the data. These codes were iteratively refined and grouped into 12 categories, which were subsequently abstracted into four overarching themes. These themes were derived inductively and were grounded in participants’ narratives rather than based on pre‐existing frameworks. Particular attention was paid to preserving the experiential and interpretative depth of each case through repeated reading, detailed memo writing, and close engagement with participants’ language. This process preceded higher‐level abstraction.

To support the organization and systematic management of qualitative data, MAXQDA 2022 software was employed. An audit trail was maintained throughout the analysis process, including coding decisions, theme development notes, and analytic memos, to ensure transparency and traceability of the findings. In addition, to enhance the credibility of the findings, member checking was conducted. A subset of participants was invited to review the preliminary themes to verify their accuracy and resonance with their original accounts. This iterative and reflective analytic approach ensured that the emergent themes closely reflected participants’ perspectives while maintaining methodological transparency and trustworthiness. Although the sample size was larger than that used in some IPA studies, efforts were made to preserve analytic depth through an idiographic, case‐by‐case approach. Each transcript was analyzed in detail before moving to the next case, and themes were first developed within individual accounts before being examined across participants for patterns of convergence and divergence. In line with IPA’s idiographic commitment, as emphasized in the IPA framework [23], each participant’s account was treated as a unique case, and individual meanings were preserved before developing shared themes across cases. This approach aimed to maintain IPA’s interpretative and idiographic focus while allowing an exploration of shared and context‐specific experiences.

### 2.7. Ethical Considerations

Ethical approval was obtained from the Istanbul University Social and Human Sciences Research Ethics Committee (date: 27.08.2024 and approval number: 2837219). Written informed consent was obtained from all participants after they were informed of the study’s aims, procedures, confidentiality measures, and their right to withdraw from the study at any time. Interviews were audio‐recorded only with participants’ permission, and all personal information was anonymized. Data confidentiality and secure storage were maintained throughout the study.

### 2.8. Rigor and Reflexivity

To ensure trustworthiness and methodological rigor, the research team employed strategies such as independent coding, peer debriefing, and reflexive journaling. Researchers had expertise in both qualitative methodology and infection control in clinical settings. To minimize potential bias, researchers engaged in continuous reflexive practice and maintained awareness of their potential influence on data interpretation. Data triangulation and member checking were used to enhance the credibility and consistency of the findings. Themes were reviewed through peer discussion and consensus to strengthen the dependability of the analysis. The combination of these strategies strengthened the trustworthiness of the research and the credibility of the conclusions drawn. In addition, the first author evaluated the reporting quality of the study using the 32‐item Consolidated Criteria for Reporting Qualitative Research (COREQ) checklist for qualitative studies incorporating in‐depth interviews (Appendix 1).

## 3. Results

A total of 15 participants were included in the study (Table 1). This study analyzed the experiences of nurses working in internal medicine clinics regarding infection control measures. These experiences were grouped under four main themes: (1) Awareness and Compliance with Infection Control and Prevention Standards, (2) Challenges in Infection Control Measure Implementation, (3) The Role of Training, and (4) Monitoring, Supervision, and Continuous Improvement. Each theme offers insight into nurses’ knowledge, attitudes, and behaviors related to infection control measures. These findings highlight both the strengths and limitations in current practices, identifying key areas for improvement and support to enhance infection control measures within healthcare settings (Table 2).

**TABLE 2:** Main themes and subthemes.

Main theme	Subtheme	Representative initial codes
Awareness and Compliance with Infection Control	Identifying Infection Risk	Awareness of infection risk, catheter infection concern, monitoring risk, patient safety responsibility
	Compliance with Standard Procedures	Hand hygiene difficulty, PPE challenges, time pressure, skipping precautions, COVID‐19 vigilance

Challenges in Infection Control Measure Implementation	Workload and Staff Shortages	Staff insufficiency, high patient load, fatigue, prioritizing tasks, inability to maintain isolation
	Physical Environment and Equipment Shortages: Deviation from Ideal Conditions	Inadequate cleaning, old infrastructure, limited space, lack of materials, unsuitable environment
	Attitudes and Behaviors of Other Healthcare Professionals	Physician noncompliance, interprofessional tension, difficulty warning others, inconsistent practices

The Role of Education	The Impact of Educational Programs: Enhanced knowledge and awareness	Increased awareness, orientation benefit, knowledge gain, repetition fatigue
	From Training to Practice: Barriers Encountered in Implementation	Theory–practice gap, ineffective online training, lack of hands‐on experience, workload barriers

Monitoring, Supervision, and Continuous Improvement	The Significance of Supervision and Feedback in Professional Practice	Audit importance, feedback need, committee role, monitoring awareness
	Sustaining Continuous Monitoring and Improvement Suggestions	Lack of regular audits, need for solutions, improvement suggestions, continuous evaluation

*Note:* Representative initial codes are presented to illustrate the analytic process; a total of 128 initial codes were generated.

### 3.1. Awareness and Compliance With Infection Control and Prevention Standards

#### 3.1.1. Identifying Infection Risk

Nurses emphasized the critical role of infection control and prevention standards in ensuring patient safety. They particularly noted their awareness of specific risks associated with intravenous (IV) infections and the use of other catheters. Nurses’ ability to accurately identify these risks may enhance their adherence to infection control measures. Their focus on high‐risk patients and clinical situations may directly influence the effectiveness of infection prevention strategies. One nurse summarized the perception of these risks by stating:

*“We are always aware of the heightened risk of infections, especially with invasive devices like IV lines and catheters. It’s our responsibility to monitor these risks continuously to ensure patient safety.” (P1)*

This account suggests that nurses perceive infection risk awareness not merely as technical knowledge but as an ongoing professional responsibility closely tied to patient safety and vigilant caregiving.

#### 3.1.2. Compliance With Standard Procedures

Nurses described hand hygiene, the use of PPE, and isolation measures as fundamental elements of infection control. They indicated that, with the onset of the COVID‐19 pandemic, they became more vigilant about adhering to these standard procedures. The increased awareness brought on by the pandemic appeared to heighten sensitivity to measures such as regular handwashing and hand disinfection. However, factors such as heavy workloads, time constraints, and the lack of readily accessible handwashing stations made it challenging for nurses to fully comply with these procedures. Some nurses, particularly in emergency situations and high‐traffic departments, noted that tasks such as washing or disinfecting hands before and after every patient could sometimes be neglected, making adherence to standard procedures difficult. One nurse expressed the practical difficulties faced as follows:

*“On a busy day, running between patients, it can sometimes be hard to put on a gown and gloves every time. For shorter tasks, we might have to skip these precautions. However, this always makes me feel uneasy because reducing the risk of infection is our primary responsibility. Since COVID-19, we′ve been more careful about these matters, but unfortunately, it′s not always possible to fully adhere to these ideals under pressure and time constraints.” (P4)*

This narrative reflects a tension between nurses’ commitment to infection control ideals and the practical pressures of everyday clinical work, revealing how compliance is experienced not only as a procedural task but also as a source of professional unease when ideal standards cannot be fully maintained.

Overall, these findings indicate that strong awareness alone does not ensure consistent implementation when routine care is shaped by time pressure and structural constraints.

### 3.2. Challenges in Infection Control Measure Implementation

In this study, nurses provided detailed accounts of the challenges they encountered while implementing infection control measures. These challenges were examined under three subthemes: Workload and Staff Shortages; Physical Environment and Equipment Shortages: Deviation from Ideal Conditions; and Attitudes and Behaviors of Other Healthcare Professionals.

#### 3.2.1. Workload and Staff Shortages

One of the main challenges in implementing infection control measures was workload and staff shortages. Nurses reported that they struggled to fully comply with infection control procedures due to high patient numbers and insufficient staffing. Some nurses mentioned that critical measures, such as contact isolation, could sometimes be neglected under the pressure of a busy working day. Faced with these difficulties, nurses were often required to reprioritize tasks, which sometimes resulted in infection control measures being overlooked. Nurses acknowledged these lapses but explained that under the burden of heavy workloads and time constraints, such situations could become difficult to avoid. One nurse summarized the situation as follows:

*“Because the ward is very busy… we have a limited number of staff. So, as they get tired… they start letting some things slide. For example, we can′t fully implement contact isolation; because putting on a gown and changing gloves for every patient each time is both time-consuming and exhausting.” (P7)*

These experiences suggest that nurses often experience a tension between professional responsibility and the practical limitations of the clinical environment, leading to feelings of frustration, moral burden, and reduced perceived control over patient safety. Thus, although nurses strive to adhere to infection control measures, heavy workloads and time pressures can make full and consistent implementation difficult, posing a significant barrier to the continuity and effectiveness of infection control.

#### 3.2.2. Physical Environment and Equipment Shortages: Deviation From Ideal Conditions

The inadequacy of the physical environment and the lack of necessary equipment were significant factors that made the implementation of infection control measures more difficult. Nurses working in older buildings emphasized that inadequate cleaning services and environments unsuitable for infection control could increase risks and reduce the effectiveness of preventive measures. In addition, inadequate training of cleaning staff and the challenges posed by older hospital buildings in terms of cleanliness were cited as other factors that could make infection control difficult. Participants felt that improving cleaning services and modernizing the working environment could help address these issues. One nurse summarized the situation as follows:

*“The rooms are small, and as the buildings are old, in terms of cleanliness… I don′t think the cleaning is very thorough. The cleaning staff aren′t meticulous either. For example, they wipe the sink with the same cloth they use to clean the table. This increases the risk of infection.” (P12)*

This finding indicates that inadequate physical conditions were perceived not merely as practical inconveniences but as structural constraints that could limit nurses’ ability to maintain safe and effective infection control practices.

#### 3.2.3. Attitudes and Behaviors of Other Healthcare Professionals

The successful implementation of infection control measures is contingent upon the collective efforts of healthcare professionals, including nurses. However, nurses highlighted that when other professionals demonstrated limited attention to infection control measures, they sometimes assumed responsibility for addressing the situation. Nevertheless, they also observed that such actions could lead to discord within the team, potentially undermining the continuity and effectiveness of infection control measures. One nurse’s statement exemplifies this challenge:

*“It is my opinion that doctors do not pay as much attention to hand hygiene as nurses do. On occasion, when we issue reminders, we encounter a variety of responses.” (P15)*

These accounts suggest that inconsistencies in infection control practices across professional groups may create interpersonal tension within the clinical team and place additional moral and professional responsibility on nurses. This dynamic may undermine teamwork, weaken adherence to standard protocols, and compromise the sustainability of infection prevention efforts.

### 3.3. The Role of Education

All nurses emphasized the critical importance of education for infection control measures. In this context, the effects of educational programs on knowledge and awareness, the practical implications of training, and the obstacles encountered were explored in detail.

#### 3.3.1. The Impact of Educational Programs: Enhanced Knowledge and Awareness

Nurses emphasized the perceived value of training on infection control measures in raising awareness and understanding the importance of standard procedures. Orientation programs, particularly for newly recruited nurses, were highlighted as crucial for learning the fundamental principles of infection control. One nurse expressed this as follows:

*“The training provided has led to an observable increase in awareness about infection control among newly-qualified nurses. The knowledge acquired during the orientation process encourages them to prioritise infection prevention, which is reflected in their practices.” (P13)*

Nevertheless, some nurses observed that repetitive training sessions could result in desensitization over time. The frequency and format of the training appeared to have varying effects on participants. This phenomenon is exemplified by the following statement from one nurse:

*“Yes, exactly. We listen, but it feels like we’re listening passively, at least that’s how I feel sometimes. It’s like I’m listening but not really absorbing the information, or maybe I already know it. You know how you can make mistakes in something you know very well? It’s kind of like that. Being exposed to constant training isn′t always pleasant. It would be better to have them at intervals.” (P8)*

Taken together, these statements suggest that nurses do not view training simply as a source of information but as a process that shapes their sense of professional competence and engagement. When training becomes repetitive and passive, it may reduce motivation and weaken the perceived relevance of infection control practices. These statements emphasize the need for a careful balance between the frequency and format of training. Nurses proposed that a more beneficial approach would involve strategically planned training sessions rather than continuous repetition, to raise awareness and convey information effectively.

#### 3.3.2. From Training to Practice: Barriers Encountered in Implementation

While training programs are critical for the effective implementation of infection control measures, several obstacles may arise when translating knowledge acquired from training into routine practice. Participants reported that factors such as heavy workloads, staff shortages, and insufficient repetition of training sessions made full compliance with infection control measures challenging. These barriers could hinder the practical application of theoretical knowledge gained during training, thereby limiting the desired effectiveness of infection control measures in the field.

Moreover, nursing professionals highlighted the perceived lower effectiveness of online training compared to face‐to‐face sessions. Although online education offers advantages in terms of time and resources, participants noted that these sessions are less interactive and lack opportunities for hands‐on learning, a key benefit of in‐person training. One nurse articulated this sentiment:

*“Online training is less effective than face-to-face sessions. In a face-to-face environment, learners have the opportunity to engage directly with the procedures. However, the practical component is missing in online training, leaving us with only theoretical knowledge.” (P3)*

This quotation highlights a perceived gap between knowing infection control principles and feeling capable of applying them confidently in practice. The limited interactivity of online training may weaken nurses’ sense of preparedness and reduce the practical value they attribute to such education. This highlights the importance of the practical aspects of training and shows how online sessions may exacerbate these challenges. Nurses emphasized that, to support the effective implementation of infection control measures, training could be more interactive and practice oriented. To enhance the practical effectiveness of training, face‐to‐face sessions could be reconsidered and prioritized over online training where appropriate. Blended approaches that combine the accessibility of online formats with the experiential depth of in‐person instruction may offer a more effective and sustainable model for infection control education in clinical settings.

### 3.4. Monitoring, Supervision, and Continuous Improvement

This section addresses the importance of monitoring, supervision, and continuous improvement processes in enhancing the effectiveness and sustainability of infection control measures. All nurses concurred that regular supervision and feedback mechanisms are of paramount importance in improving adherence to infection control measures. However, they also highlighted that these mechanisms are not adequately or effectively implemented by management in their respective institutions. This theme provides a comprehensive exploration of the significance of supervision and feedback, the challenges encountered, and potential strategies for overcoming these challenges.

#### 3.4.1. The Significance of Supervision and Feedback in Professional Practice

Nurses underscored the importance of periodic monitoring for supporting the effective implementation of infection control measures, while also highlighting the inadequacy of existing audit procedures in their institutions. They suggested that effective supervision processes, together with feedback derived from these processes, could contribute to continuous improvement in infection control practices.

Nurses placed particular emphasis on the role of infection control committees in conducting audits, which they perceived as a valuable means of reducing hospital infection rates. Furthermore, they observed that these audits serve not only to identify limitations but also to educate and raise awareness. One nurse described the dual function of these audits as follows:

*“Following an increase in the number of catheter infections in the clinic, the infection control committee undertook a review of the procedures in place. “By examining all stages, from catheter insertion to subsequent care, the committee was able to identify the errors that were being made.” (P9)*

This reflects nurses’ perception of supervision and feedback not merely as control mechanisms but as supportive processes that may strengthen professional awareness, reinforce accountability, and improve infection control practice.

#### 3.4.2. Sustaining Continuous Monitoring and Improvement Suggestions

The lack of frequent and effective monitoring and evaluation procedures was identified by nurses as a significant obstacle to the consistent implementation of infection control measures. The majority of nurses agreed that regular, solution‐oriented audits conducted by infection control committees could be more effective in reducing infection rates. It was emphasized that such audits should not only monitor the current situation but also provide recommendations for improvement, thus supporting the process. One nurse summarized the proposed approach for enhancing the effectiveness of audits as follows:

*“I believe that the audits should be more functional in nature.” (P11)*

In addition to identifying limitations, solutions should be provided to facilitate improvement. For example, the objective should be to identify methods of reducing the risk of infection in a room with seven patients.

This reflects nurses’ expectation that monitoring should be constructive and solution oriented rather than limited to identifying shortcomings. Such an approach may be important for sustaining improvement and strengthening the practical value of infection control efforts.

## 4. Discussion

This study explored the experiences of nurses working in internal medicine clinics regarding infection control measures and the challenges they faced in implementing these measures. The findings indicate that although infection control measures were highly valued and accepted by nurses, their practical implementation was hindered by various barriers.

The study found that nurses working in internal medicine clinics had a high level of awareness and knowledge of infection prevention standards. However, adherence to these standards was often inconsistent due to practical constraints. This finding is consistent with a systematic review of 18 studies involving 4577 nurses and nursing students, which showed that although nurses have sufficient knowledge of infection control, their practical application remains at an average to suboptimal level [26]. This highlights that knowledge and awareness alone may not be sufficient; rather, appropriate working conditions appear necessary to facilitate the effective implementation of infection control measures.

The study revealed that factors complicating and occasionally hindering nurses’ adherence to infection control and prevention standards included heavy workloads, staff shortages, inadequate physical environments, and the attitudes and behaviors of other healthcare professionals. There is limited qualitative research exploring nurses’ experiences with infection control measures, and no studies have specifically focused on internal medicine clinic settings. Similar findings have been reported in studies conducted with nurses in other departments, where workload, unsuitable environments, and interpersonal challenges were identified as barriers to infection control practices [17, 19]. These results emphasize that ensuring compliance may require not only individual awareness but also attention to systemic factors such as staffing, support, and infrastructure.

Nurses in this study highlighted the perceived value of face‐to‐face training in enhancing their awareness and understanding of infection control measures. Studies in the literature support this observation, showing that education can improve compliance with infection control standards [27–29]. However, there is a gap in the literature regarding the comparative effectiveness of online versus face‐to‐face training. This underscores the need for future research to determine the most effective training modalities for infection control.

Participants also identified audit, monitoring, and continuous improvement processes as important for supporting adherence to infection control measures. They noted that these processes were inadequate in their institutions, emphasizing the need for more systematic and effective implementation. This finding aligns with evidence in the literature demonstrating that audit and feedback mechanisms can promote compliance with infection control measures [30, 31]. The lack of effective auditing and monitoring systems, as identified in this study, represents a significant obstacle to effective infection control. Institutions may therefore consider strengthening these processes to enhance adherence to infection control measures and improve patient outcomes.

This study provides valuable insights into nurses’ experiences with infection control in internal medicine clinics, using an interpretative phenomenological approach. One of the study’s strengths lies in its ability to capture rich, contextualized narratives that illustrate how institutional and interpersonal factors shape infection control practices.

However, several limitations should be acknowledged. First, the study was conducted in the internal medicine units of a single university hospital, which may limit the transferability of the findings to other clinical settings with different infrastructure, staffing models, or infection control cultures. Second, the use of a phenomenological design, while valuable for capturing lived experiences, may limit direct comparison with findings derived from other methodological approaches, such as quantitative or mixed‐method studies. Third, the relatively small sample size, though sufficient for data saturation in qualitative research, may not fully reflect the diversity of experiences across broader healthcare environments. Finally, while semistructured interviews enabled participants to share their perspectives openly, the potential for subjective or socially desirable responses cannot be excluded.

## 5. Conclusion

This study suggests that while nurses maintain a high level of awareness and accountability regarding infection prevention, their ability to consistently implement infection control measures may be constrained by organizational and systemic challenges, such as staffing shortages, increased workloads, inadequate infrastructure, and limited interdisciplinary collaboration. These barriers may compromise protocol adherence and contribute to diminished job satisfaction and elevated patient risk. The findings suggest that knowledge alone may be insufficient for sustainable infection control; rather, effective implementation appears to require institutional support, adequate resources, and a workplace culture that promotes shared responsibility. Strategic improvements, such as enhanced audit systems, hands‐on and context‐sensitive training, and the inclusion of nurses in policy decision‐making, may help reinforce infection control practices in similar clinical settings.

### 5.1. Implications for Nursing Management

Nursing leaders and healthcare administrators may consider developing context‐sensitive institutional policies that address the systemic barriers identified in this study. Nurse managers may support adherence to infection control protocols by reviewing nurse‐to‐patient ratios and daily workflow demands. Hospital administrators could consider improving physical infrastructure and ensuring the availability of adequate equipment and resources where feasible. Infection control committees may contribute to practice improvement by implementing regular audit and feedback systems to monitor compliance and guide continuous improvement.

In similar internal medicine settings, attention to nurse‐to‐patient ratios, physical environments, and interdisciplinary accountability may help support infection control practices. In addition, nurse managers and infection control teams could consider simulation‐based, practice‐oriented training programs adapted to local needs and resources. Moreover, involving nurses in the planning, evaluation, and refinement of infection control policies may improve both feasibility and long‐term sustainability. Strengthening frontline engagement and leadership in infection prevention efforts may contribute to organizational safety and care outcomes.

## Author Contributions

All authors contributed to the conceptual development and theoretical analysis presented in this manuscript. The initial draft was written by Ilknur Özkan, and Cansu Polat Dunya, along with all authors, were involved in revising, editing, and refining the manuscript.

## Funding

This research did not receive any specific grant from funding agencies in the public, commercial, or not‐for‐profit sectors.

## Disclosure

All authors have reviewed and approved the final version.

## Ethics Statement

This study was approved by the Istanbul University Social and Human Sciences Research Ethics Committee under the approval number (27.08.2024‐2837219).

## Conflicts of Interest

The authors declare no conflicts of interest.

## Supporting Information

Additional supporting information can be found online in the Supporting Information section.

## Supporting information


**Supporting Information** COREQ checklist for qualitative studies incorporating in‐depth interviews.pdf.pdf.

## Data Availability

The data that support the findings of this study are available from the corresponding author upon reasonable request.

## References

[1] Kozłowski B. , Kubiak-Pulkowska J. , Pałka J. , Bożiłow D. , Zając M. , and Deptuła A. , Healthcare-Associated Infections in COVID-19 ICU Patients–Two-Centre Study, Central European Journal of Public Health. (2022) 30, no. 3, 196–200, 10.21101/cejph.a7135. 36239369

[2] Polly M. , de Almeida B. L. , Lennon R. P. , Cortês M. F. , Costa S. F. , and Guimarães T. , Impact of the COVID-19 Pandemic on the Incidence of Multidrug-Resistant Bacterial Infections in an Acute Care Hospital in Brazil, American Journal of Infection Control. (2022) 50, no. 1, 32–38, 10.1016/j.ajic.2021.09.018. 34562526 PMC8457917

[3] Gidey K. , Gidey M. T. , Hailu B. Y. , Gebreamlak Z. B. , and Niriayo Y. L. , Clinical and Economic Burden of Healthcare-Associated Infections: A Prospective Cohort Study, PLoS One. (2023) 18, no. 2, 10.1371/journal.pone.0282141. PMC9949640 36821590

[4] Ling M. L. , Apisarnthanarak A. , and Madriaga G. , The Burden of Healthcare-Associated Infections in Southeast Asia: A Systematic Literature Review and Meta-Analysis, Clinical Infectious Diseases. (2015) 60, no. 11, 1690–1699, 10.1093/cid/civ095. 25676799

[5] Mitchell B. G. , Shaban R. Z. , MacBeth D. , Wood C.-J. , and Russo P. L. , The Burden of Healthcare-Associated Infection in Australian Hospitals: a Systematic Review of the Literature, Infection, Disease and Health. (2017) 22, no. 3, 117–128, 10.1016/j.idh.2017.07.001. 31862087

[6] Thandar M. M. , Matsuoka S. , Rahman O. , Ota E. , and Baba T. , Infection Control Teams for Reducing Healthcare-Associated Infections in Hospitals and Other Healthcare Settings: A Protocol for Systematic Review, BMJ Open. (2021) 11, no. 3, 10.1136/bmjopen-2020-044971. PMC7938975 33674376

[7] Ershova K. , Savin I. , Kurdyumova N. et al., Implementing an Infection Control and Prevention Program Decreases the Incidence of Healthcare-Associated Infections and Antibiotic Resistance in a Russian Neuro-ICU, Antimicrobial Resistance and Infection Control. (2018) 7, 1–11, 10.1186/s13756-018-0383-4. 30083313 PMC6069828

[8] Castro-Sánchez E. and Holmes A. H. , Impact of Organizations on Healthcare-Associated Infections, Journal of Hospital Infection. (2015) 89, no. 4, 346–350, 10.1016/j.jhin.2015.01.012. 25726435

[9] Ferreira L. , Azevedo L. , Salvador P. , Morais S. , Paiva R. , and Santos V. , Nursing Care in Healthcare-Associated Infections: A Scoping Review, Revista Brasileira de Enfermagem. (2019) 72, no. 2, 476–483, 10.1590/0034-7167-2018-0418. 31017213

[10] Valdano E. , Poletto C. , Boëlle P.-Y. , and Colizza V. , Reorganization of Nurse Scheduling Reduces the Risk of Healthcare-Associated Infections, Scientific Reports. (2021) 11, no. 1, 10.1038/s41598-021-86637-w. PMC8016903 33795708

[11] Ahsan A. , Dewi E. S. , Suharsono T. et al., Knowledge Management-Based Nursing Care Educational Training: A Key Strategy to Improve Healthcare-Associated Infection Prevention Behavior, SAGE Open Nursing. (2021) 7, 10.1177/23779608211044601. PMC8642116 34869859

[12] Ayed A. , Malak M. Z. , Ayed M. , Allayed R. , and Shouli M. , Knowledge, Attitudes, and Practices Toward Infection Control Precautions Among Nurses in Palestinian Hospitals, International Journal of Nursing Education Scholarship. (2024) 21, no. 1, 10.1515/ijnes-2023-0117. 39428754

[13] Bawaqneh K. A. , Ayed A. , and Salameh B. , Nurses’ Knowledge, Attitude, Practice, and Perceived Barriers of Infection Control Measures in the Intensive Care Units at Northwest Bank Hospitals, Critical Care Nursing Quarterly. (2025) 48, no. 2, 160–171, 10.1097/cnq.0000000000000538. 40009862

[14] Al-Masalmah N. , Ayed A. , Batran A. , Salameh B. , and Ejheisheh M. A. , Evidence-Based Guidelines for the Prevention of Ventilator-Associated Pneumonia: Knowledge, Adherence and Perceived Barriers Among Nurses, British Journal of Healthcare Management. (2025) 31, no. 8, 1–12, 10.12968/bjhc.2025.0102.

[15] Batran R. , Ayed A. , Batran A. et al., Determinants of Nurses’ Compliance With Infection Prevention and Control Practices in Critical Care Units, SAGE Open Nursing. (2025) 11, 10.1177/23779608251339193. PMC12046164 40321410

[16] Khallaf H. , Ejheisheh M. A. , Malak M. Z. et al., Nurses’ Knowledge, Attitudes, and Decision-Making Related to Sepsis Assessment and Management in Palestinian Intensive Care Units, BMC Nursing. (2025) 24, no. 1, 10.1186/s12912-025-03341-0. PMC12211300 40598298

[17] Henderson J. , Willis E. , Roderick A. , Bail K. , and Brideson G. , Why do Nurses Miss Infection Control Activities? A Qualitative Study, Collegian. (2020) 27, no. 1, 11–17, 10.1016/j.colegn.2019.05.004.

[18] Ward D. J. , Attitudes Towards the Infection Prevention and Control Nurse: An Interview Study, Journal of Nursing Management. (2012) 20, no. 5, 648–658, 10.1111/j.1365-2834.2012.01354.x. 22823221

[19] Wendt B. , Huisman-de Waal G. , Bakker-Jacobs A. , Hautvast J. L. , and Huis A. , Exploring Infection Prevention Practices in Home-Based Nursing Care: A Qualitative Observational Study, International Journal of Nursing Studies. (2022) 125, 10.1016/j.ijnurstu.2021.104130. 34839222

[20] Binda F. , Marelli F. , Cesana V. , Rossi V. , Boasi N. , and Lusignani M. , Prevalence of Delayed Discharge Among Patients Admitted to the Internal Medicine Wards: A Cross-Sectional Study, Nursing Reports. (2025) 15, no. 3, 10.3390/nursrep15030098. PMC11944830 40137671

[21] Parker S. H. , Jesso M. N. , Wolf L. D. et al., Human Factors Contributing to Infection Prevention in Outpatient Hemodialysis Centers: A Mixed Methods Study, American Journal of Kidney Diseases. (2024) 84, no. 1, 18–27, 10.1053/j.ajkd.2023.12.024. 38447708 PMC11193600

[22] Almutairi H. A. S. , Alzahrani A. A. G. , Al Sillah H. N. et al., Infection Control Protocols for Patient Safety: Collaboration of Dentist, Medical Nurse, Medical Doctor, Radiology, Laboratory, Nutrition, Anesthesia, Pharmacy, and Medical Information Department, The Review of Diabetic Studies. (2025) 243–278.

[23] Smith J. A. , Larkin M. , and Flowers P. , Interpretative Phenomenological Analysis: Theory, Method and Research, 2021, Sage, https://www.torrossa.com/it/resources/an/5282221.

[24] Smith J. A. , Reflecting on the Development of Interpretative Phenomenological Analysis and Its Contribution to Qualitative Research in Psychology, Qualitative Research in Psychology. (2004) 1, no. 1, 39–54.

[25] Patton M. Q. , Qualitative Research & Evaluation Methods: Integrating Theory and Practice, 2014, Sage publications.

[26] Nasiri A. , Balouchi A. , Rezaie-Keikhaie K. , Bouya S. , Sheyback M. , and Al Rawajfah O. , Knowledge, Attitude, Practice, and Clinical Recommendation Toward Infection Control and Prevention Standards Among Nurses: A Systematic Review, American Journal of Infection Control. (2019) 47, no. 7, 827–833, 10.1016/j.ajic.2018.11.022. 30612817

[27] Farotimi A. A. , Ajao E. O. , Ademuyiwa I. Y. , and Nwozichi C. U. , Effectiveness of Training Program on Attitude and Practice of Infection Control Measures Among Nurses in Two Teaching Hospitals in Ogun State, Nigeria, Journal of Education and Health Promotion. (2018) 7, no. 1, 10.4103/jehp.jehp_178_17. PMC6009152 29963564

[28] Kim Y. , Kim M. Y. , and Seo Y. H. , The Effects of an Intensive Education Program on Hospital Infection Control on Nursing Students’ Knowledge, Attitude, and Confidence in Infection Control, Journal of Korean Biological Nursing Science. (2016) 18, no. 4, 318–326, 10.7586/jkbns.2016.18.4.318.

[29] Xiong P. , Zhang J. , Wang X. , Wu T. L. , and Hall B. J. , Effects of a Mixed Media Education Intervention Program on Increasing Knowledge, Attitude, and Compliance With Standard Precautions Among Nursing Students: A Randomized Controlled Trial, American Journal of Infection Control. (2017) 45, no. 4, 389–395, 10.1016/j.ajic.2016.11.006. 27986296 PMC7115275

[30] Keizer J. , Bente B. E. , Al Naiemi N. , Van Gemert-Pijnen L. J. , and Beerlage-De Jong N. , Improving the Development and Implementation of Audit and Feedback Systems to Support Healthcare Workers in Limiting Antimicrobial Resistance in the Hospital: Scoping Review, Journal of Medical Internet Research. (2022) 24, no. 3, 10.2196/33531. PMC8957011 35275082

[31] Lenglet A. , van Deursen B. , Viana R. et al., Inclusion of real-time Hand Hygiene Observation and Feedback in a Multimodal Hand Hygiene Improvement Strategy in low-resource Settings, JAMA Network Open. (2019) 2, no. 8, 10.1001/jamanetworkopen.2019.9118. PMC6694391 31411711

